# Impact of comorbidities on hospital mortality in patients with acute pancreatitis: a population-based study of 110,021 patients

**DOI:** 10.1186/s12876-023-02730-6

**Published:** 2023-03-23

**Authors:** Nils Jimmy Hidalgo, Elizabeth Pando, Rodrigo Mata, Nair Fernandes, Sara Villasante, Marta Barros, Daniel Herms, Laia Blanco, Joaquim Balsells, Ramon Charco

**Affiliations:** 1 Universitat Autonoma de Barcelona, Barcelona, Spain; 2 Department of Hepato-Pancreato-Biliary and Transplant Surgery, Hospital Universitari Vall d’Hebron, Passeig de La Vall d’Hebron, 119-129. 08035 Barcelona, Spain

**Keywords:** Acute pancreatitis, Hospital mortality, Comorbidity, Charlson index, Elixhauser index

## Abstract

**Background:**

The impact of pre-existing comorbidities on acute pancreatitis (AP) mortality is not clearly defined. Our study aims to determine the trend in AP hospital mortality and the role of comorbidities as a predictor of hospital mortality.

**Methods:**

We analyzed patients aged ≥ 18 years hospitalized with AP diagnosis between 2016 and 2019. The data have been extracted from the Spanish National Hospital Discharge Database of the Spanish Ministry of Health. We performed a univariate and multivariable analysis of the association of age, sex, and comorbidities with hospital mortality in patients with AP. The role of the Charlson and Elixhauser comorbidity indices as predictors of mortality was evaluated.

**Results:**

A total of 110,021 patients diagnosed with AP were hospitalized during the analyzed period. Hospital mortality was 3.8%, with a progressive decrease observed in the years evaluated. In multivariable analysis, age ≥ 65 years (OR: 4.11, *p* < 0.001), heart disease (OR: 1.73, *p* < 0.001), renal disease (OR: 1.99, *p* < 0.001), moderate-severe liver disease (OR: 2.86, *p* < 0.001), peripheral vascular disease (OR: 1.43, *p* < 0.001), and cerebrovascular disease (OR: 1.63, *p* < 0.001) were independent risk factors for mortality. The Charlson > 1.5 (OR: 2.03, *p* < 0.001) and Elixhauser > 1.5 (OR: 2.71, *p* < 0.001) comorbidity indices were also independently associated with mortality, and ROC curve analysis showed that they are useful for predicting hospital mortality.

**Conclusions:**

Advanced age, heart disease, renal disease, moderate-severe liver disease, peripheral vascular disease, and cerebrovascular disease before admission were independently associated with hospital mortality. The Charlson and Elixhauser comorbidity indices are useful for predicting hospital mortality in AP patients.

**Supplementary Information:**

The online version contains supplementary material available at 10.1186/s12876-023-02730-6.

## Background

Acute pancreatitis (AP) is a prevalent acute inflammatory disease that affects the pancreas, with an increased incidence in recent years [1, 2]. Most cases are mild with a self-limited course [3]. However, patients with severe acute pancreatitis have a high mortality rate (20–50%) [4–6]. For this reason, many efforts have been made to find predictors of severity and mortality in patients with AP [7–11] to identify patients who need admission to an intensive care unit or specific treatment.

In clinical practice, systems such as the Ranson score, the Acute Physiology and Chronic Health Evaluation II (APACHE II) score, the Computed Tomography Severity Index (CTSI), the Bedside Index for Severity in Acute Pancreatitis (BISAP), and various biochemical markers are used to predict severe AP and mortality [3, 12–16]. However, hospital mortality in AP could also be related to intrinsic patient characteristics, such as individual comorbidities. Most classic scores do not consider comorbidities before admission, except for APACHE II, but are restricted to severe chronic diseases.

According to some previous studies, patients with certain comorbidities, such as obesity [17], hypertriglyceridemia [18], chronic renal failure [19], diabetes [20, 21], and systemic lupus erythematosus [22], are associated with a higher risk of AP severity and mortality. However, few studies currently evaluate the impact of comorbidities on AP severity and mortality.

Our study aimed to determine the relevance of comorbidities and their indexes (Charlson and Elixhauser) as predictors of hospital mortality in patients with AP.

## Methods

### Study design

We carried out a retrospective observational study using the Spanish National Hospital Discharge (*Registro de Actividad de Atención Especializada-Conjunto Mínimo Básico de Datos, “RAE-CMBD”*). The RAE-CMBD collects all the administrative data from hospitals (public and private) in the country [23].

The information collected in this database comes from hospital discharge reports made by the physicians in charge of the patient. This information and record of coded diagnoses are automatically collected by the computer software of each center or by technical-administrative staff.

### Study population

The study population includes patients diagnosed with AP and admitted to the Spanish National Health System hospitals from 2016 to 2019. Since 2016, the RAE-CMDB has collected 20 diagnoses and 20 procedures from each patient based on the International Classification of Diseases Version 10 (ICD-10).

The inclusion criteria were: Patients with a primary or first registered secondary diagnosis of AP.

The exclusion criteria were: 1) Patients under 18 years of age, 2) Patients with a diagnosis primary or first registered secondary of pancreatic neoplasm, chronic pancreatitis, pancreatic cyst, pancreatic pseudocyst, extrahepatic bile duct neoplasm, and complications of the transplanted pancreas. We excluded the population under 18 years of age because the incidence of AP is lower in the pediatric population [24], and the etiologies distribution differs from that of adults [25].

### Variables analyzed

The variables included are demographic variables such as age and sex, and AP etiology. We used the ICD-10 diagnostic code from each patient to identify the etiology of AP, which includes six categories: biliary, alcohol, idiopathic, drug-related, other, and unspecified. Our clinical-administrative database does not have data on diagnostic tests such as ultrasound or magnetic resonance imaging that allow the identification of the etiology in patients with a diagnosis of "unspecified pancreatitis. In addition, we did not have data after hospital discharge that could expand the information on the etiology. Other variables assessed were clinical variables on diagnoses and procedures.

### Comorbidity assessment

Comorbidities were identified from the ICD-10 diagnosis codes of each patient. We have used the POA indicator (Present on registration) to identify comorbidities and differentiate them from diagnoses produced during hospital admission that could be secondary complications of AP. The ICD-10 codes used to identify specific comorbidities are described in the supplementary material (Additional file 1).

### Calculation of comorbidities indexes

We assessed comorbidity by calculating the Charlson [26] and Elixhauser [27] comorbidity indices. These two indices are used in medical practice to predict mortality. ICD-10 diagnosis codes described by Quan et al. [28] were applied to identify specific comorbidities from the Charlson and Elixhauser indices.

The Charlson index assigns weights for 17 specific diseases, and its value was calculated by adding the weights of each condition as described by Charlson et al. [26]. The Elixhauser index assigns weights for 30 specific diseases, and its value was calculated using the algorithm described by Walraven et al. [29].

### Outcomes

Outcomes analyzed included pancreatic necrosis length of hospital stay, admission to intensive care unit (ICU), length of ICU stay, and hospital mortality. Since 2018, the definition of pancreatic necrosis and pancreatic necrosis infection has been included in the ICD-10 diagnosis code for AP: AP without necrosis or infection (K85. × 0), AP with uninfected necrosis (K85. × 1) and AP with infected necrosis (K85. × 2). In addition, we did not have information on the percentage extension of pancreatic necrosis. Therefore, the data on pancreatic necrosis were only used for the descriptive analysis of the evolution of AP in the period analyzed.

### Statistical analysis

We used the Kruskal–Wallis test for continuous variables and the Linear-by-Linear association test for categorical variables to analyze the characteristics and results of patients with AP during the years evaluated (2016–2019).

The analysis of risk factors for hospital mortality was performed by applying the chi-square test for categorical variables and the Student's t-test or the Mann–Whitney U test for continuous variables.

Univariate and multivariable analysis of the factors associated with hospital mortality was performed using logistic regression. We performed three multivariable analyses of significant variables in the univariate analysis. The first analysis included: age ≥ 65 years, sex, and specific comorbidities. In the second analysis, the Charlson comorbidity index replaced the specific comorbidities. The third analysis replaced specific comorbidities with the Elixhauser comorbidity index.

Receiver-operating characteristic (ROC) curves were drawn to analyze the in-hospital mortality prediction capacity of the Charlson and Elixhauser comorbidity indices, and the area under the curve (AUC) was described. A Delong test [30] was performed to compare the AUC. We used the Youden's index to identify the best cut-off point for the Charlson and Elixhauser comorbidity indices.

Statistical analyses were performed using IBM SPSS 20.0 (IBM Corp. in Armonk, NY) and Stata version 16 (Stata, College Station, Texas, USA). Statistical significance was set at *p* < 0.05.

### Ethical aspects

Our study follows the principles of the Declaration of Helsinki for research on human beings. The data was extracted from the Spanish Ministry of Health register, which is anonymous following Spanish legislation. Identifying patients at the individual or reporting unit level with the data obtained is impossible.

## Results

### General population characteristics

**Fig. 1:**
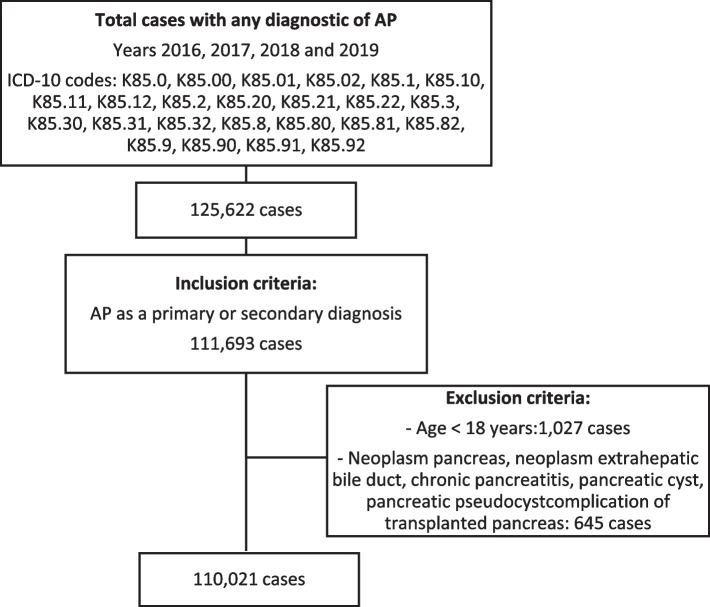
Case Selection Flow Chart. *AP: acute pancreatitis IDC-10: 10th revision of the International Statistical Classification of Diseases*

Between January 1, 2016, and December 31, 2019, a total of 125,622 cases with the diagnosis of AP were identified. After applying inclusion and exclusion criteria, 110,021 patients were included (Fig. 1). The demographic and clinical characteristics of the population and its variations during the period are shown in Table 1. The mean age was 64.32 ± 17.94 years, with a slight progressive decrease throughout the years. The 53.3% of patients were 65 years or older. Male sex prevalence was 53.1%, which significantly increased during the study period (*p* = 0.043). The most frequent etiologies of AP were biliary (41.2%) and alcohol (7.9%). The Charlson and Elixhauser comorbidity indices values progressively increased in the last years of the study period (Table 1).

**Table 1:** Characteristics of patients hospitalized with a diagnosis of acute pancreatitis

	Total (N: 110,021)	2016 (N: 26,952)	2017 (N: 22,170)	2018 (N: 29,785)	2019 (N: 31,114)	*p* value
Age, years						
Mean ± SD	64.32 ± 17.94	64.54 ± 17.99	64.34 ± 17.82	64.33 ± 17.97	64.11 ± 17.96	0.017
Age ≥ 65 years, N (%)	58,612 (53.3)	14,679 (54.5)	11,806 (53.3)	15,832 (53.2)	16,295 (52.4)	< 0.001
Sex, N (%)						
Male	58,457 (53.1)	14,178 (52.6)	11,765 (53.1)	15,899 (53.4)	16,615 (53.4)	0.043
Female	51,564 (46.9)	12,774 (47.4)	10,405 (46.9)	13,886 (46.6)	14,499 (46.6)	0.043
Charlson Index						
Mean ± SD	0.95 ± 1.47	0.88 ± 1.41	0.91 ± 1.43	0.97 ± 1.5	1.02 ± 1.53	< 0.001
Elixhauser Index						
Mean ± SD	3.43 ± 5.87	3.24 ± 5.71	3.2 ± 5.66	3.49 ± 5.93	3.72 ± 6.08	< 0.001
Pancreatitis etiology, N (%)						
Biliary	45,281 (41.2)	9,909 (36.8)	8,263 (37.3)	13,354 (44.9)	13,755 (44.2)	< 0.001
Alcohol	8,658 (7.9)	1,708 (6.3)	1,477 (6.7)	2,625 (8.8)	2,848 (9.2)	< 0.001
Medications	1,086 (1)	197 (0.7)	177 (0.8)	332 (1.1)	380 (1.2)	< 0.001
Idiopathic	2,456 (2.2)	380 (1.4)	366 (1.7)	821 (2.8)	889 (2.9)	< 0.001
Other	4,029 (3.7)	947 (3.5)	909 (4.1)	1,038 (3.5)	1,135 (3.6)	0.761
Not specified	48,511 (44.1)	13,811 (51.2)	10,978 (49.5)	11,615 (39)	12,107 (38.9)	< 0.001
Pancreatic necrosis, N (%)	-	NR	NR	2,523 (8.5)	2,873 (9.2)	
Infected necrosis, N (%)	-	NR	NR	918 (3.1)	1,069 (3.4)	
ICU admission, N (%)	5,155 (4.7)	1,187 (4.4)	1,093 (4.9)	1,445 (4.9)	1,430 (4.6)	0.383
ICU stay (days)						
Mean ± SD	13.05 ± 22.27	13.15 ± 22.05	12.65 ± 22.06	13.35 ± 21.57	13.97 ± 23.31	0.181
Hospital stay (days)						
Mean ± SD	9.38 ± 12.22	9.73 ± 12.11	9.43 ± 12.02	9.35 ± 12.35	9.08 ± 12.29	< 0.001
Mortality, N (%)	4,153 (3.8)	1,095 (4.1)	884 (4)	1,097 (3.7)	1,077 (3.5)	< 0.001

*SD* Standard deviation, *NR* Not reported, *ICU* Intensive care unit

### General outcomes

The proportion of patients who required ICU admission was 4.7%, with no differences in its prevalence by year studied. The mean length of hospital stay was 9.38 ± 12.22 days, showing a significant decrease over the period. Pancreatic necrosis was reported in 8.5% in 2018 and 9.2% in 2019 (Table 1).

### Mortality

Mortality was 3.8% in all the population and significantly decreased over time, from 4.1% in 2016 to 3.5% in 2019 (*p* < 0.001) (Table 1).

### Impact of Age, sex, and etiology

Age and male sex were higher in the non-survivors compared to the group of survivors (78.02 ± 13.24 vs. 63.78 ± 17.89, *p* =  < 0.001 and 51.1% vs. 53.2%, *p* = 0.007, respectively). The prevalence of pancreatitis of biliary, alcoholic, and drug-related etiology was lower in the group of non-survivors (*p* < 0.001) (Table 2).

**Table 2:** Demographic characteristics of acute pancreatitis according to survivor and non-survivors

	Total	Survivors	Non-survivors	*p* value
Age, mean ± SD	64.32 ± 17.94	63.78 ± 17.89	78.02 ± 13.24	< 0.001
Sex, N (%)				
Male	58,457 (53.1)	56,335 (53.2)	2,122 (51.1)	0.007
Female	51,564 (46.9)	49,533 (46.8)	2,031 (48.9)	0.007
Comorbidities, N (%)				
Arterial hypertension	51,532 (46.8)	49,011 (46.3)	2,521 (60.7)	< 0.001
Heart disease	18,696 (17)	17,202 (16.2)	1,494 (35.9)	< 0.001
Chronic pulmonary disease	9,234 (8.4)	8,739 (8.3)	495 (11.9)	< 0.001
Renal disease	8,193 (7.4)	7,399 (7)	794 (19.1)	< 0.001
Moderate or severe liver disease	1,498 (1.4)	1,375 (1.3)	123 (3)	< 0.001
Diabetes mellitus	20,259 (18.4)	19,315 (18.2)	944 (22.7)	< 0.001
Obesity	9,681 (8.8)	9,327 (8.8)	354 (8.5)	0.523
Peripheral vascular disease	3,361 (3.1)	3,091 (2.9)	270 (6.5)	< 0.001
Cerebrovascular disease	2,220 (2)	2,023 (1.9)	197 (4.7)	< 0.001
Rheumatic disease	1,335 (1.2)	1,261 (1.2)	74 (1.8)	0.001
Charlson Index, mean ± SD	0.95 ± 1.47	0.92 ± 1.43	1.76 ± 2.1	< 0.001
Elixhauser Index, mean ± SD	3.43 ± 5.87	3.29 ± 5.75	7.15 ± 7.38	< 0.001
Pancreatitis etiology, N (%)				
Biliary	45,281 (41.2)	44,041 (41.6)	1,240 (29.9)	< 0.001
Alcohol	8,658 (7.9)	8,533 (8.1)	125 (3)	< 0.001
Medications	1,086 (1)	1,070 (1)	16 (0.4)	< 0.001
Idiopathic	2,456 (2.2)	2,333 (2.2)	123 (3)	0.001
Other	4,029 (3.7)	3,768 (3.6)	261 (6.3)	< 0.001
Not specified	48,511 (44.1)	46,123 (43.6)	2,388 (57.5)	< 0.001
ICU admission, N (%)	5,155 (4.7)	3,684 (3.5)	1,471 (35.4)	< 0.001

*SD* Standard deviation, *ICU* Intensive care unit

### Impact of comorbidities and indexes

Non-survivor patients presented a higher percentage in all comorbidities except for obesity (Table 2).

Median values of Charlson and Elixhauser indexes were significantly higher in the group of non-survivors compared with survivors (1.76 ± 2.1 vs. 0.92 ± 1.43, *p* < 0.001 and 7.15 ± 7.4 vs. 3.29 ± 5.8, *p* < 0.001 respectively) (Table 2). The analysis of the comorbidities included in the Charlson and Elixhauser indices according to hospital mortality is described in the supplementary material (Additional file 1).

### Multivariable analysis

Logistic regression analysis was performed using the best cut-off point obtained by Youden's index (*J* = 1.5 points for both Charlson and Elixhauser indices). After multivariable logistic regression analysis, we found that factors independently associated with mortality were age 65 years or older (OR 4.11, 95% CI 3.75–4.5), heart disease (OR 1.73, 95% CI 1.62–1.86), renal disease (OR 1.99, 95% CI 1.74–2.07), moderate-severe liver disease (OR 2.86, 95% CI 2.35–3.47), peripheral vascular disease (OR 1.43, 95% CI 1.25–1.64), and cerebrovascular disease (OR 1.63, 95% CI 1.4–1.9). Arterial hypertension has been found to be a protective factor in the population. The Charlson Index > 1.5 points (OR 2.03, 95% CI 1.9–2.16) and Elixhauser Index > 1.5 points (OR 2.71, 95% CI 2.53–2.9) were independently associated with mortality (Table 3).

**Table 3:** Multivariable analysis showing association of proposed risk factors with hospital mortality in acute pancreatitis

	Multivariable analysis	
	OR (95% CI)	*p* value
Variables of analysis 1		
Age ≥ 65 years	4.11 (3.75–4.5)	< 0.001
Sex Female	0.96 (0.9–1.03)	0.236
Arterial hypertension	0.89 (0.84–0.96)	0.003
Heart disease	1.73 (1.62–1.86)	< 0.001
Chronic pulmonary disease	1.09 (0.99–1.21)	0.085
Renal disease	1.99 (1.74–2.07)	< 0.001
Moderate or severe liver disease	2.86 (2.35–3.47)	< 0.001
Diabetes mellitus	0.94 (0.87–1.01)	0.09
Peripheral vascular disease	1.43 (1.25–1.64)	< 0.001
Cerebrovascular disease	1.63 (1.4–1.9)	< 0.001
Rheumatic disease	1.13 (0.89–1.44)	0.317
Variables of analysis 2		
Age ≥ 65 years	4.41 (4.04–4.8)	< 0.001
Sex Female	0.96 (0.9–1.03)	0.962
Charlson Index ≥ 1.5	2.03 (1.9–2.16)	< 0.001
Variables of analysis 3		
Age ≥ 65 years	4.23 (3.88–4.61)	< 0.001
Sex Female	0.98 (0.92–1.05)	0.581
Elixhauser Index ≥ 1.5	2.71 (2.53–2.9)	< 0.001

*OR* Odds ratio, *CI* Confidence interval

### AUC analysis

**Fig. 2:**
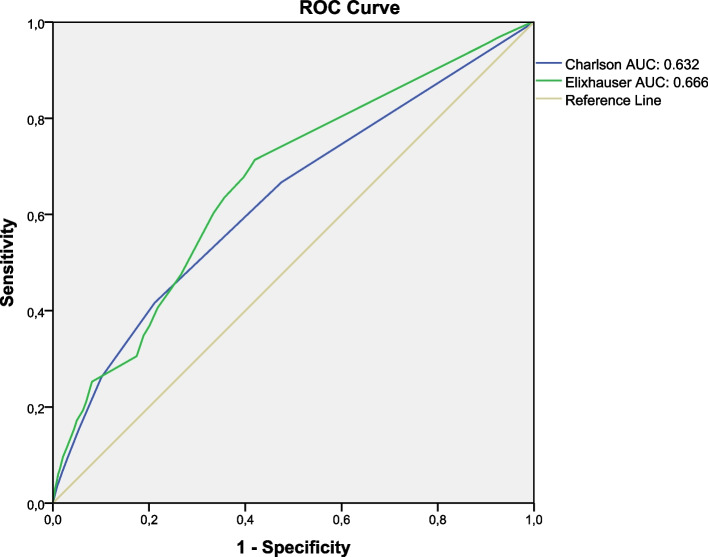
ROC curve and AUC (Area Under the Curve) of the Charlson Comorbidity Index, and the Elixhauser Comorbidity Index in predicting the hospital mortality rate in patients with acute pancreatitis

The Elixhauser comorbidity index exhibited a slightly higher AUC value in predicting hospital mortality (AUC: 0.666, 95% CI 0.657 – 0.674) than the Charlson comorbidity index (AUC: 0.633, 95% CI 0.623 – 0.641). When performing the Delong test to compare these AUC, it was observed that this difference is significant (*p* < 0.001). The ROC curves and AUC for the Charlson and Elixhauser comorbidity indices to predict hospital mortality are shown in Fig. 2.

## Discussion

Our study found that pre-admission comorbidities such as heart disease, kidney disease, moderate-severe liver disease, peripheral vascular disease, cerebrovascular disease, and age ≥ 65 years were independently associated with mortality in AP. Charlson and Elixhauser comorbidity indices were independently associated with mortality.

Advanced age has been extensively studied as a marker of severity and mortality in AP. Most studies report longer hospitalization [31, 32] and higher overall mortality from AP in elderly patients [33–36]. However, other studies have observed that older patients may have a more severe course of AP but do not present increased mortality [37]. Likely explanations explaining advanced age as a risk factor for mortality include a proinflammatory state in older people [38] and increased production of cytokines in elderly patients with sepsis [39]. Other reasons would be delayed diagnosis and treatment due to less clinical and analytical expression [40, 41].

The increase in life expectancy and the aging of the population have been associated with the increase in patients with comorbidities [42, 43], so determining its impact on AP becomes more necessary. The importance of comorbidities in predicting outcomes in other diseases that require acute hospital admission is well known [44–46]. However, few studies analyze the impact of comorbidities on severity and mortality in AP patients [47, 48]. Additionally, few studies have incorporated comorbidities in their clinical models when evaluating determinants of AP severity. Our study is one of the first studies in the literature that put in relevance the role of comorbidities in AP.

In the last period of our study, we observed an increase in comorbidities and the values of the Charlson and Elixhauser comorbidity indices. These trends could be explained by the increase in life expectancy and the prevalence of chronic diseases in the European population in the last decades [49, 50]. However, despite the increase in comorbidities, hospital mortality has decreased in the period studied. The decrease in mortality is likely due to the advances in critical care medicine, step-up approach to treat infected necrosis, and surgical and endoscopic new approaches [3, 51].

Few studies had previously assessed comorbidity indexes' role in predicting mortality in patients with AP. Previous studies have observed that more comorbidities are associated with organ failure and mortality in patients with AP [47, 52]. In our study, we analyzed the Charlson and Elixhauser indices which are good predictors of mortality in other diseases [53, 54]. Our results showed that values > 1.5 points for both indices are independently associated with hospital mortality in AP after adjusting for age and sex. Future studies that expand knowledge of the effects of comorbidities on complications and mortality in patients with AP will improve the identification of patients at risk and their quality of care.

Regarding other comorbidities, our results align with previous studies stating that pre-existing heart and renal diseases predict mortality in patients with AP [19, 47, 55]. One hypothesis is that intravascular depletion and aggressive fluid resuscitation cause decompensation of previous heart and renal disease [55].

Similarly, to Frey et al. [47], we found an association between liver disease, peripheral vascular disease, and cerebrovascular disease with mortality. However, Murata et al. found no association between these diseases with mortality [55]. The worse results of AP in patients with liver diseases such as cirrhosis could be explained because they present an inflammatory syndrome with arterial vasodilation and release of proinflammatory cytokines that increase the severity of AP [56]. In addition, acute pancreatitis produces significant stress that could decompensate underlying chronic comorbidities and increase the risk of death.

Other major comorbidities before admission, such as chronic lung disease, were not independently associated with mortality, coinciding with the results of Murata et al. [55]. Similar controversial results were found regarding obesity, in which previous studies have observed that obesity is a risk factor for developing local and systemic complications and mortality in patients with AP [17, 57–59]. We did not find an association between hospital mortality and obesity, but this result has to be taken carefully due to the potential information bias because our results are based on the history of obesity recorded in the medical-administrative database and not on the BMI at hospital admission. In the same line, we did not find a relation between diabetes mellitus and mortality after the multivariable analysis. The literature remains controversial, with reports describing diabetes mellitus as a risk factor for mortality [20, 21]. However, Frey et al. found that diabetes increased the risk of multiple organ failure but was not associated with mortality [47].

Other risk factors classically related to mortality in AP patients failed to represent a risk factor in our population. This was relevant to the role of AP etiology, in which the literature reports controversial results, identifying a more severe course and higher mortality in alcoholic pancreatitis [58, 59]. In contrast, others observed greater severity in biliary pancreatitis [60, 61] or no relation with mortality between both aetiologies [37, 62–64]. Our study did not observe that acute pancreatitis's biliary or alcoholic etiology was associated with higher hospital mortality. However, in our study, 44.3% of the patients were classified as "unspecified acute pancreatitis,” limiting the precision of our results and constituting a bias regarding the real impact of etiology in AP mortality. In addition, our database does not include other etiologies of acute pancreatitis such as those caused by hypercalcemia, after trauma, viral infections, anatomical variants, iatrogenic after endoscopic retrograde cholangiopancreatography or endoscopic ultrasound-guided interventions [65–67].

Our study is subject to some limitations. The data analysis from a clinical-administrative base has low level of granularity and does not include some clinical results of interest, such as severity or the evolution of the patient in the medium or long term after their hospital stay. Our study could not identify pancreatic necrosis in the first two years because it began to be considered in the ICD-10 in 2018. Another limitation is the potential underreporting of information because the hospital discharge report may be incomplete or poorly registered by the technical-administrative staff. In our study, we could not identify the etiology of AP in many patients due to a lack of precise coding.

No studies have been published to validate the use of ICD-10 codes for identifying patients with AP using the RAE-CMBD database. However, there are recent studies from Danish [68] and US [69] databases with PPV of 97.3% and 87%, respectively. A recent meta-analysis recommends using ICD codes only in incident cases of AP in adults, where it reaches a PPV of 78% [70]. However, this may be because the studies analyzed used ICD-8, ICD-9, and ICD-10 codes, and the PPV is higher when using the ICD-10 because it is more specific and includes the etiology of pancreatitis [70, 71]. In addition, the studies were carried out in different hospitals in several countries, contributing to the heterogeneity.

We used the primary and the first registered secondary diagnoses to reduce the bias of not including patients with an initial diagnosis of cholelithiasis/choledocholithiasis and AP. A recent study validating ICD codes using primary and secondary diagnoses observed a PPV of 97.3% for AP [68].

The main strength of our study is its large sample size which provides strong statistical power. The RAE-CMBD database is a mandatory registry for the Spanish National Health System, which covers almost 100% of admissions in Spain, reinforcing the external validity of our results. In addition, the database has several internal audit mechanisms and has proven its usefulness for health research [53, 72, 73].

## Conclusions

Comorbidities such as heart disease, kidney disease, moderate-severe liver disease, peripheral vascular disease, cerebrovascular disease, and advanced age were independently associated with mortality in AP. The Charlson and Elixhauser comorbidity indices are useful for predicting hospital mortality in these patients**.**

## Supplementary Information

**Additional file 1:** **Table S1.** The ICD-10 codes used to identify specific comorbidities. **Table S2.** Association of comorbidities of Charlson Comorbidity Index and hospital mortality in acute pancreatitis. **Table S3.** Association of comorbidities of Elixhauser Comorbidity Index and hospital mortality in acute pancreatitis.

## Data Availability

The datasets used and analyzed during the current study are available from the corresponding author on reasonable request.

## Abbreviations

AP: Acute pancreatitis
APACHE II: Acute physiology and chronic health evaluation II
CTSI: Computed tomography severity index
BISAP: Bedside index for severity in acute pancreatitis
RAE-CMBD: Registro de Actividad de Atención Especializada-Conjunto Mínimo Básico de Datos
ICD-10: International classification of diseases version 10
POA: Present on registration
ICU: Intensive care unit
ROC: Receiver-operating characteristic
AUC: Area under the curve
OR: Odds ratio
CI: Confidence interval
