# Supplementary material for: Impact of comorbidities on hospital mortality in patients with acute pancreatitis: a population-based study of 110,021 patients
Source: BMC Gastroenterol. 2023 Mar 23;23:81. doi: 10.1186/s12876-023-02730-6 (PMC10035222; doi:10.1186/s12876-023-02730-6)
Supplement: Supplementary file 1 — Additional file 1: Table S1. The ICD-10 codes used to identify specific comorbidities. Table S2. Association of comorbidities of Charlson Comorbidity Index and hospital mortality in acute pancreatitis. Table S3. Association of comorbidities of Elixhauser Comorbidity Index and hospital mortality in acute pancreatitis. [file 12876_2023_2730_MOESM1_ESM.docx]

Table S1. The ICD-10 codes used to identify specific comorbidities

|  | Charlson comorbidity index  ICD-10 codes | Elixhauser comorbidity index  ICD-10 codes |
| --- | --- | --- |
| Arterial hypertension |  | Hypertension, uncomplicated: I10.x  Hypertension, complicated: I11.x - I13.x, I15.x |
| Heart disease | Myocardial infarction: I21.x, I22.x, I25.2  Congestive heart failure: I09.9, I11.0, I13.0, I13.2, I25.5, I42.0, I42.5 - I42.9, I43.x, I50.x, P29.0 | Congestive heart failure: I09.9, I11.0, I13.0, I13.2, I25.5, I42.0, I42.5 - I42.9, I43.x, I50.x, P29.0  Cardiac arrhythmias: I44.1 - I44.3, I45.6, I45.9, I47.x - I49.x, R00.0, R00.1, R00.8, T82.1, Z45.0,  Z95.0  Valvular disease: A52.0, I05.x - I08.x, I09.1, I09.8, I34.x - I39.x, Q23.0 - Q23.3, Z95.2 - Z95.4 |
| Chronic pulmonary disease | Chronic pulmonary disease: I27.8, I27.9, J40.x - J47.x, J60.x - J67.x, J68.4, J70.1, J70.3 | Chronic pulmonary disease: I27.8, I27.9, J40.x - J47.x, J60.x - J67.x, J68.4, J70.1, J70.3 |
| Renal disease | Renal disease: I12.0, I13.1, N03.2 - N03.7, N05.2 - N05.7, N18.x, N19.x, N25.0, Z49.0 - Z49.2,  Z94.0, Z99.2 | Renal failure: I12.0, I13.1, N18.x, N19.x, N25.0, Z49.0 - Z49.2, Z94.0, Z99.2 |
| Liver disease | Mild liver disease: B18.x, K70.0 - K70.3, K70.9, K71.3 - K71.5, K71.7, K73.x, K74.x, K76.0, K76.2  - K76.4, K76.8, K76.9, Z94.4  Moderate or severe liver disease: I85.0, I85.9, I86.4, I98.2, K70.4, K71.1, K72.1, K72.9, K76.5,  K76.6, K76.7 | Liver disease: B18.x, I85.x, I86.4, I98.2, K70.x, K71.1, K71.3 - K71.5, K71.7, K72.x - K74.x,  K76.0, K76.2 - K76.9, Z94.4 |
| Diabetes mellitus | Diabetes without chronic complication: E10.0, E10.1, E10.6, E10.8, E10.9, E11.0, E11.1, E11.6,  E11.8, E11.9, E12.0, E12.1, E12.6, E12.8, E12.9, E13.0, E13.1, E13.6, E13.8, E13.9, E14.0,  E14.1, E14.6, E14.8, E14.9  Diabetes with chronic complication: E10.2 - E10.5, E10.7, E11.2 - E11.5, E11.7, E12.2 - E12.5,  E12.7, E13.2 - E13.5, E13.7, E14.2 - E14.5, E14.7 | Diabetes, uncomplicated: E10.0, E10.1, E10.9, E11.0, E11.1, E11.9, E12.0, E12.1, E12.9, E13.0,  E13.1, E13.9, E14.0, E14.1, E14.9  Diabetes, complicated: E10.2 - E10.8, E11.2 - E11.8, E12.2 - E12.8, E13.2 - E13.8, E14.2 - E14.8 |
| Obesity |  | Obesity: E66.x |
| Peripheral vascular disease | Peripheral vascular disease: I70.x, I71.x, I73.1, I73.8, I73.9, I77.1, I79.0, I79.2, K55.1, K55.8,  K55.9, Z95.8, Z95.9 | Peripheral vascular disorders: I70.x, I71.x, I73.1, I73.8, I73.9, I77.1, I79.0, I79.2, K55.1, K55.8,  K55.9, Z95.8, Z95.9 |
| Cerebrovascular disease | Cerebrovascular disease: G45.x, G46.x, H34.0, I60.x - I69.x |  |
| Rheumatic disease | Rheumatic disease: M05.x, M06.x, M31.5, M32.x - M34.x, M35.1, M35.3, M36.0 | Rheumatoid arthritis/collagen vascular diseases: L94.0, L94.1, L94.3, M05.x, M06.x, M08.x,  M12.0, M12.3, M30.x, M31.0 - M31.3, M32.x - M35.x, M45.x, M46.1, M46.8, M46.9 |

Table S2. Association of comorbidities of Charlson Comorbidity Index and hospital mortality in acute pancreatitis.

|  | Total | Survivors | Non-survivors | P value | OR (95% CI) |
| --- | --- | --- | --- | --- | --- |
| Myocardial infarction, N (%) | 3,095 (2.8) | 2,920 (2.8) | 175 (4.2) | <0.001 | 1.55 (1.32-1.81) |
| Congestive heart failure, N (%) | 4,648 (4.2) | 4,043 (3.8) | 605 (14.5) | <0.001 | 4.29 (3.92-4.71) |
| Peripheral vascular disease, N (%) | 3,361 (3.1) | 3,091 (2.9) | 270 (6.5) | <0.001 | 2.31 (2.03-2.63) |
| Cerebrovascular disease, N (%) | 2,220 (2) | 2,023 (1.9) | 197 (4.7) | <0.001 | 2.56 (2.2-2.97) |
| Dementia, N (%) | 2,609 (2.4) | 2,320 (2.2) | 289 (7) | <0.001 | 3.34 (2.94-3.79) |
| Chronic pulmonary disease, N (%) | 9,234 (8.4) | 8,739 (8.3) | 495 (11.9) | <0.001 | 1.5 (1.37-1.66) |
| Rheumatic disease, N (%) | 1,335 (1.2) | 1,261 (1.2) | 74 (1.8) | 0.001 | 1.51 (1.19-1.91) |
| Peptic ulcer disease, N (%) | 801 (0.7) | 749 (0.7) | 52 (1.3) | <0.001 | 1.78 (1.34-2.36) |
| Mild liver disease, N (%) | 16,916 (15.4) | 16,543 (15.6) | 373 (9) | <0.001 | 0.53 (0.48-0.58) |
| Moderate or severe liver disease, N (%) | 1,498 (1.4) | 1,375 (1.3) | 123 (3) | <0.001 | 2.32 (1.92-2.79) |
| Diabetes without chronic complication, N (%) | 18,921 (17.2) | 18,069 (17.1) | 852 (20.5) | <0.001 | 1.25 (1.16-1.36) |
| Diabetes with chronic complication, N (%) | 15,539 (1.4) | 1,428 (1.3) | 111 (2.7) | <0.001 | 2.01 (1.65-2.44) |
| Hemiplegia or paraplegia, N (%) | 202 (0.2) | 184 (0.2) | 18 (0.4) | <0.001 | 2.5 (1.54-4.06) |
| Renal disease, N (%) | 8,191 (7.4) | 7,398 (7) | 793 (19.1) | <0.001 | 3.14 (2.89-3.41) |
| Any malignancy, N (%) | 3,957 (3.6) | 3,597 (3.4) | 360 (8.7) | <0.001 | 2.69 (2.41-3.02) |
| Metastatic solid tumor, N (%) | 1,193 (1.1) | 1,033 (1) | 160 (3.9) | <0.001 | 4.07 (3.43-4.82) |
| AIDS/HIV, N (%) | 327 (0.3) | 318 (0.3) | 9 (0.2) | 0.331 | 0.72 (0.37-1.39) |

*OR: odds ratio, CI: conﬁdence interval.*

Table S3. Association of comorbidities of Elixhauser Comorbidity Index and hospital mortality in acute pancreatitis.

|  | Total | Survivors | Non-survivors | P value | OR (95% CI) |
| --- | --- | --- | --- | --- | --- |
| Congestive heart failure, N (%) | 4,648 (4.2) | 4,043 (3.8) | 605 (14.6) | <0.001 | 4.29 (3.92-4.71) |
| Cardiac arrhythmias, N (%) | 12,043 (10.9) | 11,054 (10.4) | 989 (23.8) | <0.001 | 2.68 (2.49-2.89) |
| Valvular disease, N (%) | 4,231 (3.8) | 3,919 (3.7) | 312 (7.5) | <0.001 | 2.11 (1.88-2.38) |
| Pulmonary circulation disorders, N (%) | 1,145 (1) | 1,018 (1) | 127 (3.1) | <0.001 | 3.25 (2.69-3.92) |
| Peripheral vascular disease, N (%) | 3,361 (3.1) | 3,091 (2.9) | 270 (6.5) | <0.001 | 2.32 (2.03-2.63) |
| Hypertension, N (%) | 51,532 (46.8) | 49,011 (46.3) | 2,521 (60.7) | <0.001 | 1.79 (1.68-1.91) |
| Paralysis, N (%) | 198 (0.2) | 180 (0.2) | 18 (0.4) | <0.001 | 2.56 (1.57-4.15) |
| Other neurological disorders, N (%) | 3,415 (3.1) | 3,140 (3) | 275 (6.6) | <0.001 | 2.32 (2.04-2.64) |
| Chronic pulmonary disease, N (%) | 9,234 (8.4) | 8,739 (8.3) | 495 (11.9) | <0.001 | 1.5 (1.37-1.66) |
| Diabetes, uncomplicated, N (%) | 17,531 (15.9) | 16,756 (15.8) | 775 (18.7) | <0.001 | 1.22 (1.13-1.32) |
| Diabetes, complicated, N (%) | 2,813 (2.6) | 2,636 (2.5) | 177 (4.3) | <0.001 | 1.74 (1.49-2.04) |
| Hypothyroidism, N (%) | 7,014 (6.4) | 6,766 (6.4) | 248 (6) | 0.279 | 0.92 (0.82-1.06) |
| Renal failure, N (%) | 8,171 (7.4) | 7,380 (7) | 791 (19) | <0.001 | 3.14 (2.89-3.41) |
| Liver disease, N (%) | 17,573 (16) | 17,041 (16.1) | 532 (12.8) | <0.001 | 0.77 (0.69-0.84) |
| Peptic ulcer disease, excluding bleeding, N (%) | 599 (0.5) | 574 (0.5) | 25 (0.6) | 0.608 | 1.11 (0.74-1.66) |
| AIDS/HIV, N (%) | 327 (0.3) | 318 (0.3) | 9 (0.2) | 0.331 | 0.72 (0.37-1.39) |
| Lymphoma, N (%) | 391 (0.4) | 360 (0.3) | 31 (0.7) | <0.001 | 2.2 (1.53-3.19) |
| Metastatic cancer, N (%) | 1,193 (1.1) | 1,033 (1) | 160 (3.9) | <0.001 | 4.07 (3.43-4.82) |
| Solid tumour without metastasis, N (%) | 3,214 (3.3) | 2,915 (2.8) | 299 (7.2) | <0.001 | 2.74 (2.42-3.1) |
| Rheumatoid arthritis/collagen vascular diseases, N (%) | 1,783 (1.6) | 1,699 (1.6) | 84 (2) | 0.036 | 1.26 (1.01-1.57) |
| Coagulopathy, N (%) | 1,971 (1.8) | 1,672 (1.6) | 299 (7.2) | <0.001 | 4.84 (4.26-5.49) |
| Obesity, N (%) | 9,681 (8.8) | 9,327 (8.8) | 354 (8.5) | 0.523 | 0.97 (0.86-1.08) |
| Weight loss, N (%) | 2,286 (2.1) | 2,086 (2) | 200 (4.8) | <0.001 | 2.52 (2.17-2.92) |
| Fluid and electrolyte disorders, N (%) | 3,885 (3.5) | 3,125 (2.9) | 760 (18.3) | <0.001 | 7.36 (6.75-8.03) |
| Blood loss anaemia, N (%) | 305 (0.3) | 283 (0.3) | 22 (0.5) | 0.002 | 1.99 (1.29-3.07) |
| Deficiency anaemia, N (%) | 3,166 (2.9) | 3,025 (2.9) | 141 (3.4) | 0.042 | 1.19 (1.01-1.42) |
| Alcohol abuse, N (%) | 17,776 (16.2) | 17,417 (16.5) | 359 (8.6) | <0.001 | 0.48 (0.43-0.54) |
| Drug abuse, N (%) | 2,024 (1.8) | 1,986 (1.9) | 38 (0.9) | <0.001 | 0.48 (0.35-0.67) |
| Psychoses, N (%) | 495 (0.4) | 465 (0.4) | 30 (0.7) | 0.007 | 1.65 (1.14-2.39) |
| Depression, N (%) | 5,234 (4.8) | 5,047 (4.8) | 187 (4.5) | 0.432 | 0.94 (0.81-1.09) |

*OR: odds ratio,* *CI:* *conﬁdence interval.*
